# Similarities between Line Fishing and Baited Stereo-Video Estimations of Length-Frequency: Novel Application of Kernel Density Estimates

**DOI:** 10.1371/journal.pone.0045973

**Published:** 2012-11-29

**Authors:** Timothy J. Langlois, Benjamin R. Fitzpatrick, David V. Fairclough, Corey B. Wakefield, S. Alex Hesp, Dianne L. McLean, Euan S. Harvey, Jessica J. Meeuwig

**Affiliations:** 1 The UWA Oceans Institute, The University of Western Australia, Perth, Western Australia, Australia; 2 School of Plant Biology, The University of Western Australia, Perth, Western Australia, Australia; 3 Centre for Marine Futures and School of Animal Biology, The University of Western Australia, Perth, Western Australia, Australia; 4 Western Australian Fisheries and Marine Research Laboratories, Department of Fisheries, Government of Western Australia, Perth, Western Australia, Australia; 5 Centre for Fish and Fisheries Research, School of Biological Sciences and Biotechnology, Murdoch University, Perth, Western Australia, Australia; University of Glasgow, United Kingdom

## Abstract

Age structure data is essential for single species stock assessments but length-frequency data can provide complementary information. In south-western Australia, the majority of these data for exploited species are derived from line caught fish. However, baited remote underwater stereo-video systems (stereo-BRUVS) surveys have also been found to provide accurate length measurements. Given that line fishing tends to be biased towards larger fish, we predicted that, stereo-BRUVS would yield length-frequency data with a smaller mean length and skewed towards smaller fish than that collected by fisheries-independent line fishing. To assess the biases and selectivity of stereo-BRUVS and line fishing we compared the length-frequencies obtained for three commonly fished species, using a novel application of the Kernel Density Estimate (KDE) method and the established Kolmogorov–Smirnov (KS) test. The shape of the length-frequency distribution obtained for the labrid *Choerodon rubescens* by stereo-BRUVS and line fishing did not differ significantly, but, as predicted, the mean length estimated from stereo-BRUVS was 17% smaller. Contrary to our predictions, the mean length and shape of the length-frequency distribution for the epinephelid *Epinephelides armatus* did not differ significantly between line fishing and stereo-BRUVS. For the sparid *Pagrus auratus*, the length frequency distribution derived from the stereo-BRUVS method was bi-modal, while that from line fishing was uni-modal. However, the location of the first modal length class for *P. auratus* observed by each sampling method was similar. No differences were found between the results of the KS and KDE tests, however, KDE provided a data-driven method for approximating length-frequency data to a probability function and a useful way of describing and testing any differences between length-frequency samples. This study found the overall size selectivity of line fishing and stereo-BRUVS were unexpectedly similar.

## Introduction

Fish length-frequency information can be used to gain an understanding of the biology and ecology of fish populations [1], [2], [3]. Biological parameters such as growth rate [4], maturity [5], functional sex for hermaphroditic species [6] and reproductive output [7] are all related to body length. This variable also correlates with catchability for a range of sampling gears such as traps [8], trawls [9] and longlines [10], and influences trophic interactions through size specific predator-prey relationships [11], [12], [13]. Length-frequency information can therefore provide additional information to compliment age data used for fish stock assessments [14], [15] and studies of the ecological effects of fishing [16].

**Figure 1:**
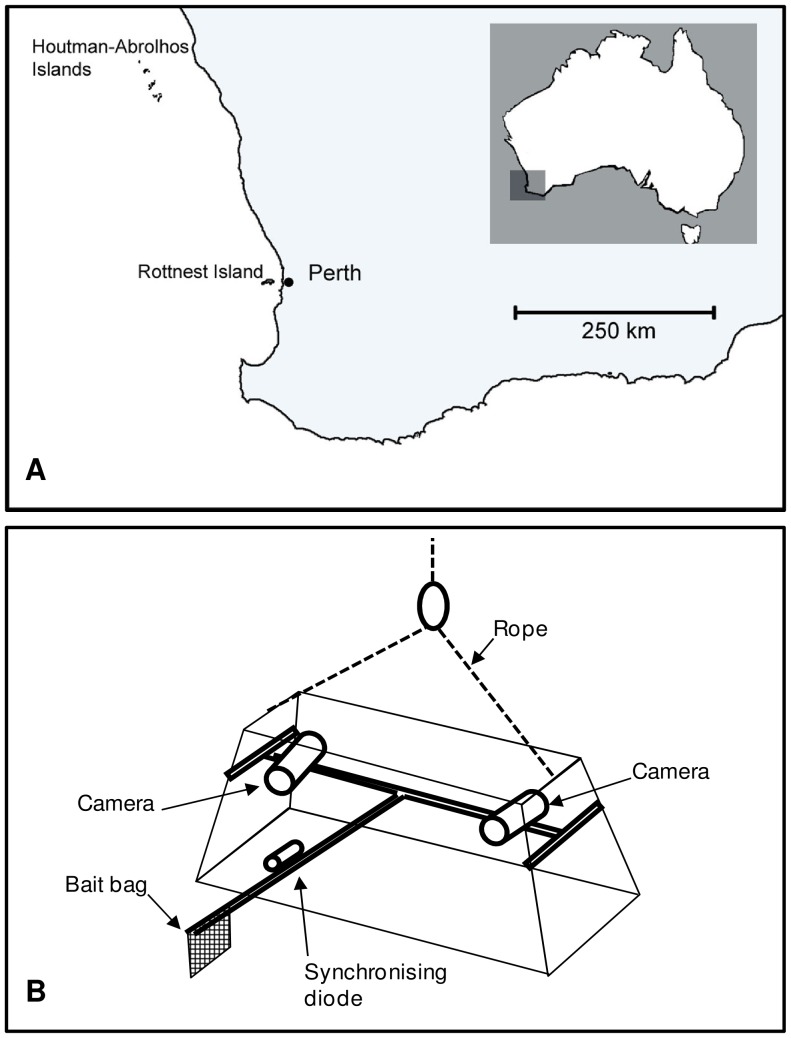
Map of Western Australia, showing the sampling locations at (A) the Houtman-Abrolhos Islands and Rottnest Island, adjacent to the Perth metropolitan area. (B) Forward-facing baited remote underwater stereo-video system (stereo-BRUVS).

As ecosystem-based approaches to fisheries management are adopted around the globe (EBFM, [17], [18]), it is becoming increasingly important to understand the predator-prey relationships of fished, by-catch and unfished species that could result in changes to assemblage composition. Studies of these interactions would benefit from using methods that sample a representative range of species from different feeding guilds and trophic levels. Forward-facing baited remote underwater stereo-video (Fig. 1b, stereo-BRUVS) is a method that samples a wide range of fish species from a variety of trophic levels, including carnivorous, herbivorous and planktivorous fishes [19]. In addition to providing estimates of diversity and abundance [20], accurate length estimates of every fish sampled can be generated from stereo-BRUVS systems [21], [22]. These data have been shown to be useful for examining temporal trends in length-frequency data of an exploited coral reef species (*Lethrinus miniatus*) over several years inside and outside protected areas [23]. Outside of protected areas stereo-BRUVS have been successfully used to correlate the abundance and biomass of exploited species across gradients in fishing pressure [24], and describe the consistent abundance distributions of endemic fish species across the old, climatically buffered seascape of south western Australia which provides a model system for biogeographic studies [25]. However, it is likely the stereo-BRUVS method has very different relative biases and selectivities to traditional fisheries dependent and independent sampling methods [20].

In south-western Australia, the majority of the age and length structure data used for the assessment and management of the exploited demersal fish species are derived from fisheries-dependent line caught samples [26], [27].We investigated the relative biases and selectivities of stereo-BRUVS compared with fisheries-independent rod and line sampling for three exploited teleosts, baldchin groper *Choerodon rubescens* (Labridae, Günther 1862) and breaksea cod *Epinephelides armatus* (Epinephelidae, Castelnau 1855), which are both endemic to the west coast of Australia, and the more widespread snapper *Pagrus auratus* (Sparidae, Bloch & Schneider 1801), which occurs across the southern half of Australia and northern New Zealand [28].

By comparing methods for sampling the length-frequency of fish populations, useful insights can be made into the particular biases and the selectivities of these methods relative to each other [20], [29], [30]. Understanding the true length-frequency of a population may be impossible as all methods, including rotenone stations, suffer from a tendency to under sample small individuals [15], although in fisheries monitoring studies it is more important to employ a standardised methodology, with standardised biases, than attempt to define the true length-frequency [31]. For example, it has been shown that a bias towards larger individuals can occur with line fishing, which may be due to dominance behaviours between fish of different size [32] and experimental studies have demonstrated that larger hook sizes tend to catch larger individuals [33]. Comparison of diver-based underwater visual census sampling (UVC) and long-lining has suggested that UVC can underestimate the mean length of some fished populations [34], [35]. In contrast, a downward-facing single-camera BRUV method has been found to collect information on relative abundance and length of a fished species (*P. auratus*) that is comparable to experimental line fishing [35]. Whilst, a forward-facing single-camera BRUV method has been shown to sample greater numbers of juvenile *P. auratus* in coastal embayment nursery areas, than experimental trapping [36]. A recent study that compared standard commercial baited fish traps and forward-facing stereo-BRUVS, found the baited video systems sampled greater abundance and size range of target and by-catch species [37]. However, the mean sizes of species, in particular target species, were comparable. Within the Stereo-BRUVS method, the mean size of fished species sampled has been found to be smaller at the time of first arrival compared to the time of maximum abundance (MaxN, [38]).

The length compositions of a teleost species can vary markedly among study sites and regions, particularly if a species is known to migrate between habitats at a certain life cycle stage or time of year. For example, the snapper *P. auratus* has been found to use large nearshore marine embayments as spawning and nursery areas throughout their distribution [39]. On the lower west coast of Australia, *P. auratus* form spawning aggregations in Cockburn and Warnbro Sounds during the Austral spring/summer [40]. Juveniles of *P. auratus* remain within these areas for about 18 months, after which they move into deeper waters, which is reflected in an increase in age and length with distance from the embayments [39]. This pattern is contrasted with the annual migration of the larger mature fish back into these embayments during the spawning season. However, many other teleost species spawn in small groups at numerous locations throughout their range, with settled juveniles to adult life stages sympatric in high relief and nearby marginal reef systems, such as *C. rubescens* [41] and *E. armatus* [42].

When comparing two sets of length-frequency data, dissimilarities can occur in both the relative location (mean length) and shape of the distribution represented by differences in mode or median relative to the mean (i.e. skewness and kurtosis, [43]). Differences in mean length of the distributions will indicate an overall bias towards either smaller or larger individuals while differences in shape indicate a particular bias towards a certain length class. Statistical methods previously used to compare single species length-frequency data fall into two main groups, those that compare mean or median length (e.g. Student's t-test or ANOVA, [35], [44]), and those that test the greatest difference between cumulative frequencies (i.e. Kolmogorov–Smirnov test, [22]). Comparisons of mean length often require powerful transformations to account for heterogeneity of variance that may, in fact, be due to differences in the shape of the distributions. In contrast, the widely used Kolmogorov–Smirnov (KS) test provides a non-parametric approach for comparing length structures via the single greatest point-difference between cumulative length-frequency distributions and is sensitive to both differences in shape and location [32], [45].

As an alternative to the KS test, kernel density estimates (KDEs) provide a data-driven method for approximating length-frequency data with probability density functions [46]. Like the KS test, KDEs also provide a non-parametric approach to compare pairs of length-frequency distributions via a permutation test for shape and location [47]. However, without KDEs, the representation of length-frequency data is reliant on histograms with bin-sizes chosen arbitrarily or via bootstrapping from very large independent samples (>1000, [48]). Sanvicente-Añorve et al. [49] first presented the use of KDEs to identify modes in length-frequency distributions and to examine their change over time. In the current study, we compare KS and KDE approaches for testing differences in distribution of length-frequency data that may be due to differences in location and/or shape. We predict the KDE approach will be more sensitive to differences between length-frequencies, given that it compares the area between two probability density functions, rather than the point difference used by the KS test.

The aims of the current study were to compare length-frequency distributions collected by a forward-facing stereo-BRUVS with those collected by line fishing for three exploited species of teleosts. Given that line fishing has a likely selectivity towards larger individuals [32] and forward-facing stereo-BRUVS has a suspected bias for a higher abundance of smaller fishes (Pers. Obs. Langlois), we hypothesised that, for each species, stereo-BRUVS would produce length-frequency distributions with relatively smaller mean lengths (location) and relatively skewed towards smaller fishes compared to line fishing.

## Methods

### Sampling regime

Length data for *C. rubescens* were obtained by 333 stereo-BRUVS deployments conducted on or adjacent to reef in waters of 5–25 m at the Houtman Abrolhos Islands during April and May of 2004, 2005 and 2006 (Fig. 1a). Length data from line fishing for *C. rubescens* were provided from the study of Fairclough [50], who collected samples from the same waters and depths in October/November 2002 (Table 1). *Epinephelides armatus* and *P. auratus* length data were obtained from 356 stereo-BRUVS deployments conducted on or adjacent to rocky reef in waters 5–80 m deep around Rottnest Island (Fig. 1a) during the Austral spring of 2007, 2008 and 2009 (September and October). Length data from line fishing for *E. armatus* and *P. auratus* were provided from the studies of Moore et al. [42] and Wakefield [51] respectively, who collected fish from the same waters and depths during all months of the year in 2003, 2004 and 2005 (Table 1). These data do not cover the full depth ranges of any of these species but do represent depths in which these species are commonly fished [42], [50], [51]. The fork length (FL) of each individual sampled by either line fishing or stereo-video was measured to the nearest 1 mm.

**Table 1:** Minimum legal length of retention (MLL), length-frequency mean and standard deviation (SD) and mode, estimated by the kernel density estimate (KDE) for *Choerodon rubescens*, *Epinephelides armatus* and *Pagrus auratus* sampled by fishing (Line) and baited remote underwater stereo-video (stereo-BRUVS).

		*Choerodon rubescens*	*Epinephelides armatus*	*Pagrus auratus*
	MLL	400	300	400
Sampling method				
Line	Mean (SD)	375 (85.1)	299 (70.9)	402 (88.7)
	ModeKDE	400	276	379
	Count	170	264	431
	Min.	179	166	202
	Max.	601	498	776
stereo-BRUVS	Mean (SD)	351 (88.7)	282 (68.8)	479 (146.9)
	ModeKDE	331	281	382
	Count	366	198	557
	Min.	133	123	177
	Max.	738	510	930

Count of lengths (Count),minimum (Min.) and maximum (Max.) lengths are also given. Values considered to illustrate differences between the length-frequency distributions are shown in bold. All fish lengths are fork length and measured in mm.

### Line fishing

Samples of *C. rubescens* were collected during a research sampling trip where fish less than the minimum legal length were allowed to be retained. Fish were typically caught using size 3/0 or 4/0 hooks and coral prawns were used as bait. Samples of *E. armatus* included fish caught during research sampling trips where fish of all lengths were allowed to be retained and by charter boat operators where a researcher was on-board to ensure samples were representative and to collect undersize fish. Fish had typically been caught employing size 3/0 or 4/0 hooks, using predominantly pilchards (*Sardinops sagax*) and squid as bait. Line fishing surveys for *P. auratus* were undertaken by research staff onboard research vessels or recreational charter vessels that were allowed to retain fish less than the minimum legal length. On each sampling occasion, a variety of rig types (from 1/0 to 7/0 hooks) and baits (predominantly pilchards and squid) were used in order to maximise the length range of fish caught [52]. The three studies from which line fishing length-frequency data was obtained [42], [50], [51] were individual biological assessments where line fishing was used to obtain a size-range of specimens, however the frequency of capture with different hook sizes was not recorded.

### Baited remote underwater stereo-video

**Deployment:** Detailed information on the design and photogrammetric specifics are presented in Harvey and Shortis [53], [54]. In brief, stereo-BRUVS systems each comprised two Sony CX12 high-definition (1920×1080) video cameras in waterproof housings mounted 0.7 m apart on a base bar. Cameras were inwardly converged at seven degrees to gain an optimized field of view, with stereo-coverage from 0.5 m in front of the cameras outwards to the maximum water visibility (Fig. 1b).

Each stereo-BRUVS was baited with 800 g of pilchards in a plastic-coated wire mesh basket, suspended 1.2 m in front of the two cameras. The pilchards were crushed to maximise bait plume dispersal. In each region, either a commercial fishing vessel or fisheries research vessel, both designed to retrieve rock lobster traps, were used to deploy the stereo-BRUVS systems. During sampling, up to 10 stereo-BRUVS systems were deployed at any one time and each left to film on the sea floor for a period of one hour. Previous research in temperate south-western Australia has found that >36 minutes is required to sample the abundance and length of the majority of fish species, and that, for including fished species, 60 minutes is advisable [19]. Adjacent replicate stereo-BRUVS samples were separated by at least 250 m to avoid overlap of bait plumes and reduce the likelihood of fish moving between stereo-BRUVSs within the same sampling period [55].

### Image analysis

The analysis of stereo-BRUVS samples was facilitated through the program EventMeasure (www.seagis.com.au). This program enabled us to manage data collected from the field operations and tape readings, capture the timing of events and reference images of the seafloor and fish in the field of view. To avoid making repeated length measurements of the same individuals, measures were made at the time of MaxN, i.e. the time when the maximum number of individuals of each species was observed at one time during the recording. Estimates of MaxN are considered conservative, particularly in areas where fish occur in high-densities [19]. The program PhotoMeasure (www.seagis.com.au) was then used to measure length from stereo-video images (snout to fork length; FL). The stereo-video techniques enable accurate length measurements to be obtained from a fish at any angle in the field of view, as long as the snout and fork can be distinguished [53]. The software calculates both distance from the cameras and length at the same time and the minimum visibility recorded was six metres. To ensure high measurement accuracy and precision, as well as a standardized sampling unit, all measures of fish length for stereo-BRUVS samples were limited to fish within a maximum distance of six metres from the cameras, resulting in a sample unit area of 37.2 m^2^.

### Statistical analyses

Length data for each species, either collected by line fishing or stereo-BRUVS, were pooled across years to produce length-frequency distributions. All species were pooled across the same number of years for each method. Length-frequency histograms were made using length classes selected to best match the shape of the probability densities generated by the kernel density estimates (KDEs). The statistical methods used are sensitive to difference in both the shape and location of length-frequency distributions. Therefore, to investigate differences due to shape alone, length-frequency data were also analysed standardised by median and variance (y = x-median/stdev), as suggested by Bowman and Azzalini [47]. All analyses were conducted using the R language for statistical computing [56].

**Kolmogorov–Smirnov test:** The Kolmogorov-Smirnov (KS) two-sample test was used to compare the two length frequency samples for each species from line fishing and stereo-BRUVS via a non-parametric test of the significance of the greatest difference in their respective cumulative distributions [57]. We used Monte Carlo simulations to overcome uncertainty regarding the asymptotic distributions of KS test statistics under the null hypothesis [58], which also enabled the test to be conducted with data containing ties. This procedure was implemented using the ‘ks.boot’ function (100,000 simulations) in the package ‘Matching’ [59].

**Figure 2:**
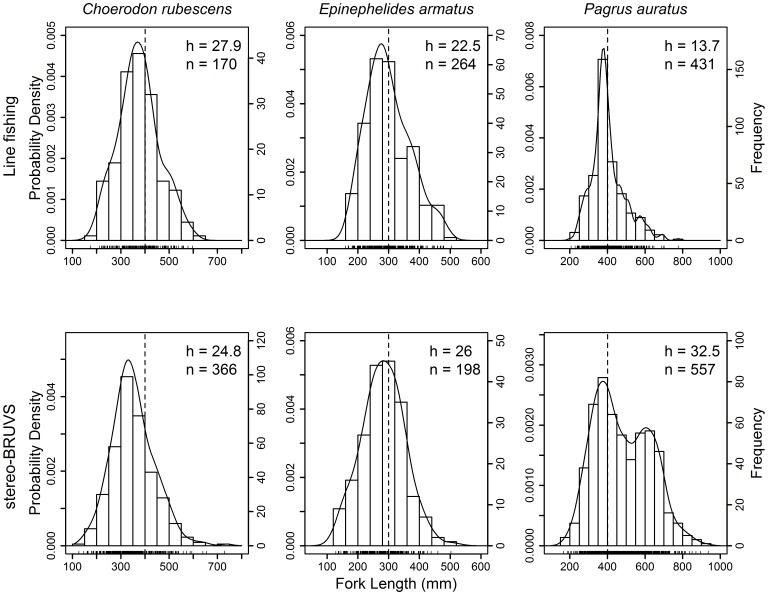
Length-frequency distribution for *Choerodon rubescens*, *Epinephelides armatus* and *Pagrus auratus* sampled using either line fishing (Line) or stereo-BRUVS. The separate bandwidth ‘h’ of each KDE was chosen by the Sheather & Jones (1991) bandwidth selection procedure. Length classes for the histogram of each species were chosen to match the KDE; *C. rubescens* 50 mm, *E. armatus* 40 mm and *P. auratus* 50 mm. Rug plot just above the x-axis indicate individual length observations. Dashed vertical lines indicate the minimum legal length of retention for each species and ‘n’, indicates the sample size.

**Kernel density estimates:** Separate KDEs were constructed for the length-frequency data for each species derived from either line fishing or stereo-BRUVS (Fig. 2). Bandwidths were selected via a ‘plug-in’ style data-driven bandwidth selection process [46], which is well-suited to univariate analyses where assumptions are not made about the nature of the distribution being estimated [2]. To check for over parameterization, the procedure of Liao et al. [60] was used, but in each case the bandwidth selected by the Sheather-Jones selection procedure was retained. If a single distribution was being approximated with a KDE, then a more advanced method, such as variable bandwidths, would be appropriate. Sheather-Jones bandwidths were estimated with the ‘dpik’ function in the package ‘KernSmooth’ [61].

The statistical test between the pairs of length-frequency distributions collected by each sampling method, for each species, was based on a null model of no difference and a permutation test. To construct the test, the geometric mean between the bandwidths for line fishing and stereo-BRUVS data were calculated for each species. This avoids the effect of differences in sample size adding more weight to the data from one method [47]. The mean bandwidths for each species were then used to construct KDEs for both the line fishing and stereo-BRUVS data. If line fishing and stereo-BRUVS data represented the same distribution, the KDEs should only differ in minor ways due to within population variance and sampling effects. The statistical test compared the area between the pair of KDEs, for line fishing and stereo-BRUVS data, to that resulting from permutations of the data into random pairs. Here, we have adapted examples given in Chapter 6 of Bowman and Azzalini [47] and implemented statistical tests using the function ‘sm.density.compare’ (100,000 permutations) in the package ‘sm’ [62].

The ‘sm.density.compare’ function also produces a plot to accompany each test with a grey band, representing the null model of no difference between the pair of KDEs. This grey band is centred on the mean KDE and extends one standard error above and below, thereby indicating which regions of the length-frequency distribution are likely to be causing any significant differences [62]. Computer code for implementing these methods and example datasets are provided (see Text S1 and Data S1).

## Results

### Description of length-frequency distributions

Length-frequency distributions of *C. rubescens* and *E. armatus* sampled by line fishing and stereo-BRUVS were similar, each with a single mode at or just below the minimum legal length (Table 1, Fig. 2). The first modes in the length compositions of *P. auratus* sampled by line fishing and stereo-BRUVS were also very similar, both occurring just below the minimum legal length (Table 1, Fig. 2). However, the length-frequency distribution of *P. auratus* sampled with stereo-BRUVS was bimodal, with a secondary mode in the histogram and KDE at a larger length (600 mm) that was not represented in the line fishing data. For all three species, stereo-BRUVS detected a wider range of lengths, which may be attributed to the larger sample size collected for *C. rubescens* and *P. auratus* using the stereo-BRUVS, however, a larger sample size was obtained by line fishing for *E. armatus* (Table 1). For *C. rubescens*, the mode of the stereo-BRUVS data was 69 mm (17.3%) smaller than that from line fishing, but there was less than 5 mm (2%) difference between the major modes of each method for *E. armatus* and *P. auratus*.

### Statistical comparison of length-frequency distributions

**Figure 3:**
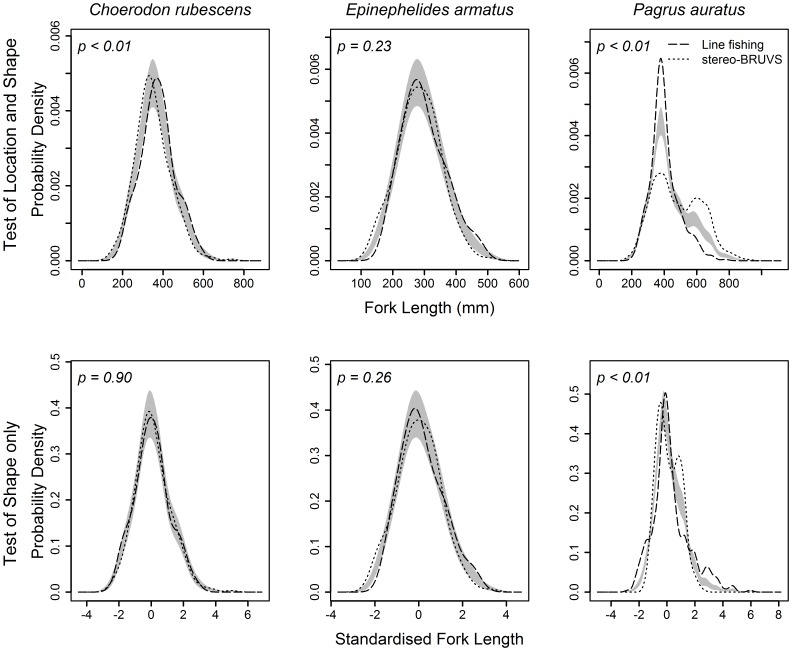
Comparison of kernel density estimate (KDE) probability density functions, using mean bandwidths, for *Choerodon rubescens*, *Epinephelides armatus* and *Pagrus auratus* sampled using either line fishing (Line) or stereo-BRUVS. Dashed and dotted lines represent the kernel density estimate (KDE) probability density functions that approximate the Line and stereo-BRUVS length-frequency data, respectively. Grey bands represent one standard error either side of the null model of no difference between the KDEs for each method. Significance tests (*p*) were based on permutation tests of the area between the two probability density functions. Significance tests on raw data (top row) provide a test of differences in both location and shape of the length-frequency distributions, whereas tests on standardised data (bottom row) provide a test of shape only.

For each species, there was consistent agreement between the statistical tests used (KS and KDE two sample tests) to compare the length-frequency distributions derived from line fishing and stereo-BRUVS (Table 2). For *C. rubescens*, both the KS and the KDE tests of shape and location found length-frequency sampled by stereo-BRUVS had a smaller mean length than line fishing (Table 2). This is illustrated by the KDE function of each sampling method lying outside the standard error band representing the null model of no difference (Fig. 3). However, once data were standardised, the test of shape found no difference in the length-frequency distributions for *C. rubescens* collected by either line fishing or stereo-BRUVS.

**Table 2:** Results of Kolmogorov–Smirnov (KS) and kernel density estimate (KDE) tests of differences between pairs of fish length-frequency distributions sampled by line fishing and baited remote underwater stereo-video.

	*Choerodon rubescens*		*Epinephelides armatus*		*Pagrus auratus*	
Statistical test	Location and Shape	Shape only	Location and Shape	Shape only	Location and Shape	Shape only
KS	**0.002**	0.950	0.130	0.393	**<0.001**	**0.001**
KDE	**<0.010**	0.900	0.230	0.260	**<0.010**	**<0.010**

Overall tests of raw data are sensitive to differences in location and shape, whereas comparison of data standardised by median and variance provide a test of shape only (Shape). *p* values are given, with significant effects (*p*<0.05) also indicated in bold.

No significant differences were detected in either the shape or location of *E. armatus* length-frequency data collected by either line fishing or stereo-BRUVS (Table 2, Fig. 3). However, significant differences were found between the location and shape of the *P. auratus* length-frequency data, with the distributions derived from line fishing and stereo-BRUVS being unimodal and bimodal, respectively (Table 2). These differences are illustrated by the deviations from the null model, standard error band, of the pair of KDE for *P. auratus* and likely to be driven by the second mode of larger fish sampled only by stereo-BRUVS (Fig. 3).

## Discussion

We found an unexpected similarity between length-frequency distributions derived from stereo-BRUVS and fishery-independent line fishing surveys of three exploited teleosts. Contrary to our hypothesis, there was no evidence of a particular bias or skew towards smaller fishes with stereo-BRUVS relative to line fishing. However, as hypothesised, the length-frequency distribution obtained for *C. rubescens* by stereo-BRUVS had a smaller mean length (17%) relative to line fishing but no particular relative bias towards smaller fish. For *E. armatus*, there were no differences in either the location or shape of the length-frequency distributions sampled by either line fishing or stereo-BRUVS. Large differences were observed in the length-frequency distributions of *P. auratus*, but these were driven by a second mode of larger fish that were sampled by stereo-BRUVS but not fishing, whereas the first mode of fish sampled by both methods was comparable (line fishing = 379 mm, stereo-BRUVS = 382 mm, Table 1). These results were unexpected, not only because of the predicted relative biases between the methods but also because of the variation in hook size and bait used for line fishing and the differences in the years of sampling between the two methods. This study found that, in addition to collecting information on a wide range of unfished species [20], [22], stereo-BRUVS provided estimates of length-frequency distributions for three exploited teleosts comparable to those sampled by fishery-independent line fishing.

The large differences between the shape of the length-frequency distributions for *P. auratus* obtained by line fishing (unimodal) and stereo-BRUVS (bimodal) suggest that large differences exist either between the methods or sampling programs used. It is thus relevant that, although both data sets were collected over the same habitats around Rottnest Island, stereo-BRUVSs were sampled during spring, whereas line fishing was conducted throughout the year. Wakefield et al. [39] has documented both temporal and spatial habitat partitioning of *P. auratus* in this region, with respect to life stage and spawning period. That study demonstrated that the age and length of *P. auratus* increased with distance offshore, until maturity when fish either spread along the shelf of the west coast or are found in particular embayment's within spawning aggregations during late spring/early summer (e.g. Cockburn Sound, adjacent to the waters sampled by the current study). The relatively higher abundance of larger fish corresponding to the second of the two modes of the KDE for *P. auratus* detected by the stereo-BRUVSs matches the mode of those associated with these spawning aggregations [39], and is thus likely to represent the pre-spawning migration of mature fish. Unfortunately, there were insufficient line data from this time of year to provide a direct comparison, and thus further surveys would be required to confirm this situation. These differences highlight the importance of considering the life history strategy of species when interpreting such data and designing sampling regimes. One would expect the length-frequency distribution of a fished population to change from year to year due to variability in recruitment, fishing pressure and variability in resources [31]. However, with temporal and spatial stratification of sampling to ensure data are representative of the different length classes in the overall population, it appears that stereo-BRUVS can provide robust estimates of length-frequency for *P. auratus*.

Although it was predicted that KDEs would provide a more sensitive test of differences in length-frequency than the established KS test, we found that the results of both approaches were comparable. Kernel density estimates (KDEs) do, however, provide a data-driven method for representing length-frequency compositions [46], instead of using histograms with length classes chosen arbitrarily or via bootstrapping from very large independent samples [48]. Although the significance test provided by KDEs is a test of overall differences, the graphical representation of the KDEs and the null model can be used to infer which regions of the length-frequency data may be responsible for any overall differences. We have found KDEs to be comparable to the established Kolmogorov–Smirnov test and provide a useful way of describing and testing differences in length-frequency data.

Particular biases and selectivities in representing the length-frequency of populations of fished species would be expected to exist for both methods considered in this study. For example, the use of a typical hook size in line fishing surveys may result in a bias against both very small [30] and very large individuals [63], depending on the size of hook used and the feeding behaviour of the species. Stereo-BRUVS, however, is likely to be able to measure both these very small and very large individuals if they approach within the field of view of the cameras. Indeed, for every species compared in this study, stereo-BRUVS did measure a slightly greater range of both small and large individuals (Table 1), however these length observations come from the tails of the distributions where observations were rare. Furthermore, forward-facing BRUV methods are used to monitor the relative abundance of small juvenile *P. auratus* (<200 mm and 2 years of age) in Cockburn Sound, Western Australia (Pers. Com. Wakefield), demonstrating that when used in nursery habitats, such a method is also applicable to obtaining length data on small fish. Given the similarities in the length distributions sampled, at least in the case of *E. armatus* and *C. rubescens*, the results of this study suggest that unexpectedly the overall biases and selectivity of fishery-independent line fishing and stereo-BRUVS were similar.

In addition to providing length distributions of these fished species, the same stereo-BRUVS deployments used in this study also collected length and abundance information from a wide range of species from a variety of trophic levels (1399 individuals from 118 taxa at Rottnest and 7065 individuals from 151 taxa from the Houtman-Abrolhos Islands). The time in the field to collect these samples was comparable to line-fishing surveys but the time in the laboratory to analyse the videos and generate the length information ranged from 2–4 hours per deployment. Although such data will be much more costly than line fishing surveys targeting particular species, this large amount of assemblage size-frequency information will be very useful for research and risk assessments directed at informing ecosystem-based approaches to fisheries management [24], [64].

Fishery-dependent line caught samples would likely have a different length distribution to those derived from fishery-independent or stereo-BRUVS, with the former restricted to fish greater than the minimum legal length. Where data for monitoring and assessment of exploited species are derived from fishery-dependent line caught fish, a stereo-BRUVS approach may provide length-based information on year class strength for a particular species ahead of recruitment to the fishery. Importantly, this study suggests that, where appropriate, stereo-BRUVS methods can provide robust length frequency data that could complement age and length data collected by line fishing surveys for single species stock assessments.

## Supporting Information

Text S1 **R code for implementing KDE methods.**
(R) Click here for additional data file.

Data S1 **Example dataset for KDE methods.**
(CSV)
